# Variation in patterns and volumes of injuries admitted to a level one trauma center during lockdown for COVID-19

**DOI:** 10.1186/s13018-022-03151-z

**Published:** 2022-06-11

**Authors:** Cornelia Ower, Kerstin Stock, Peter Kaiser, Hanno Ulmer, Rohit Arora, Matthias Haselbacher

**Affiliations:** 1 Department of Orthopaedics and Traumatology, Medical University of Innsbruck, Anichstr. 35, 6020 Innsbruck, Austria; 2 Department of Medical Statistics, Informatics and Health Economics, Medical University Innsbruck, Innsbruck, Austria; 3 Medical Treatment Facility, German Federal Armed Forces, Mittenwald, Germany

**Keywords:** COVID-19, SARS-CoV-2, Trauma center, Pandemic, Lockdown, Ischgl

## Abstract

**Introduction:**

Due to the global COVID-19 pandemic, a ban on sports outside one’s home and a prohibition on travel between communities were imposed in spring 2020 in Tyrol, Austria. The aim of this study was to evaluate the impact of these restrictions on a level one trauma center. The objective was to identify the most common injury patterns to ensure targeted prevention in times of an ongoing pandemic.

**Material and methods:**

Patients who presented themselves to our trauma center between weeks 7 and 22 in 2020 were retrospectively compared to a mean of the patients of the three previous years (2017–2019). The evaluated variables were the number of patients, age, gender, country of residence, place of accident, time of treatment, injured body region and anatomical structure, number of surgical intervention and severely injured patients.

**Results:**

Comparing the mean count of treated patients per week in 2020 of the pre-lockdown period (*n* = 804.6) with the lockdown period (*n* = 201.8) a decrease in admissions by 69.7% could be observed. The admission incidence was 9.9 times higher in previous years than in 2020 during the lockdown period. Among the injuries treated during the lockdown the largest increase in relative numbers was in home injuries, head or face injuries and superficial or penetrating injuries. There was a decrease of seriously injured patients as well as patients that needed surgery during the lockdown compared to previous years.

**Conclusions:**

We observed a significant change in the pattern and volume of injuries during a strict lockdown. Intervention programs to reduce the risk of home injuries should be introduced. Furthermore, in order to save resources during a pandemic, specific guidelines on patient management and treatment should be established for the respective medical specialties.

*Trial registration*: 1157/2020, 10.12.2020.

## Background

On March 11, 2020, the World Health Organization (WHO) classified the outbreak of the severe acute respiratory syndrome coronavirus 2 (SARS-CoV-2) in 114 countries, with a combined total of 118,000 cases, as a pandemic [1]. In Austria the very first cases of COVID-19 were confirmed in Innsbruck, Tyrol, on February 25, 2020 (week 9). In March 2020, incidents in Ischgl, Tyrol, contributed to the spread of COVID-19 across Europe. Several lockdown restrictions were implemented over the following weeks [2]. From March 16 people were not allowed to leave their home without reasonable excuse. On March 17, 2020 (week 12), the restrictions were expanded. All restaurants, bars and clubs had to remain closed. Extraordinary restrictions were implemented for the federal state of Tyrol. Quarantine was imposed from March 18 to April 07 (weeks 12–15) on all Tyrolean communities. It was forbidden to leave one’s municipality and to practice sports outside one’s own residence. In the University Hospital of Innsbruck elective surgeries had to be postponed in order to assure resources.

After April 07 (week 16) the lockdown restrictions were gradually lifted. Outdoor sports were allowed again, sports facilities could open from May 02. Stores, schools and hotels were allowed to reopen by the end of May (week 22).

Because of the lockdown in Tyrol during the global pandemic, the work routine in the traumatology department changed dramatically. Not only did the total number of admittances decrease significantly, but also injury patterns shifted. Therefore, resources had to be redistributed.

The primary aim of this study is to investigate to what extent injury patterns, place of injury and time of treatment as well as severity of injuries changed in a time of a strict lockdown as compared to the normal living conditions throughout earlier years. As a consequence of the changing nature and volume of injuries, we also aimed to evaluate the impact on the number of surgical interventions. The purpose of this study is to provide information on the number and type of injuries during a global SARS-CoV-2 pandemic. This may help to establish prevention programs for future times of crisis.

## Methods

A retrospective data analysis was conducted on all patients who presented themselves at our level one trauma center department between weeks 7 to 22 in the years 2017, 2018, 2019 and 2020. If the same patient visited the emergency service because of a second or further accident, each clinical visit was counted as a different case. The study was approved by the institutional review board (1157/2020). Data were categorized by weeks starting on Sundays. Three periods where compared: pre-lockdown (weeks 7–11), lockdown (12–15) and post-lockdown (16–22) periods.

An automatic chart search was conducted in our patient history software KIS PowerChart (“Millenium”, Cerner Corp. North Kansas City, USA). The total number of patients who presented themselves at our traumatology department in the aforementioned years, their age, gender, country of residence, the place where the injury occurred, the time of injury and the surgical intervention count were recorded. Only primary injuries were evaluated. This excluded injuries described as suspected diagnoses, secondary diagnoses or preexisting condition according to the Abbreviated Injury Scale (AIS). Up to seven injuries were evaluated per patient.

Gender was classified as male or female. The country of residence was classified as Austria, Germany or Other Countries. The place of accident was classified into five categories: at home, at work, in road traffic, at a nursing home or others (e.g., injuries in the mountain area, injuries caused by animals). The time of injury was divided into three categories: daytime from 6am–6 pm, evening (6 pm–23.59 pm) and night (0 pm–6am). The body region of the injury was classified into the categories: head and face, thorax and spine, upper and lower extremity and others. Adapted from the AIS, the injured anatomical structure was classified into the categories: superficial and penetrating, organs and muscles and tendons and ligaments, skeletal and joints and others. The injury severity was classified according to the AIS and New Injury Severity Scale (NISS) [3].

The body region of the injury and the injured anatomical structure were coded with Microsoft Excel 2016 (Microsoft, Redmond, Washington, USA).

All parameters from the lockdown period 2020 were compared to the years 2017–2019. The data were analyzed using SPSS Statistics for Windows, Version 26 (IBM Corporation, Armonk, New York, USA). P-values < 0.05 were considered to indicate statistical significance. Sociodemographic and other characteristics were compared between the lockdown period in 2020 versus the respective weeks in 2017–2019 with descriptive statistics and the use of statistical significance testing such as the Mann–Whitney U-test and the chi-square test, depending on the variable type and distribution. In addition, a series of Poisson regression models were fitted in the framework of generalized linear modes as log-linear regressions with log link and Poisson error distribution [4]. With these models, relative risks and their 95% confidence intervals were estimated for weekly admission to the trauma center for the years 2017, 2018 and 2019 versus the reference year 2020 (lockdown) in different settings (male versus female, place of injury and time of treatment). The exponents of the estimated regression coefficients were equal to the incidence rate ratio or the relative risk.

## Results

A total of 47,590 patients were treated at the trauma center of the University Hospital of Innsbruck in the weeks 7 to 22 of the years 2017–2020. Of those, 8430 cases were treated in 2020, 12,772 in 2019, 13,318 in 2018 and 13,070 in 2017. A total of 55,409 injuries were evaluated. Of those, 10,432 injuries were evaluated in 2020, 14,539 in 2019, 15,313 in 2018 and 15,125 in 2017.

**Fig. 1:**
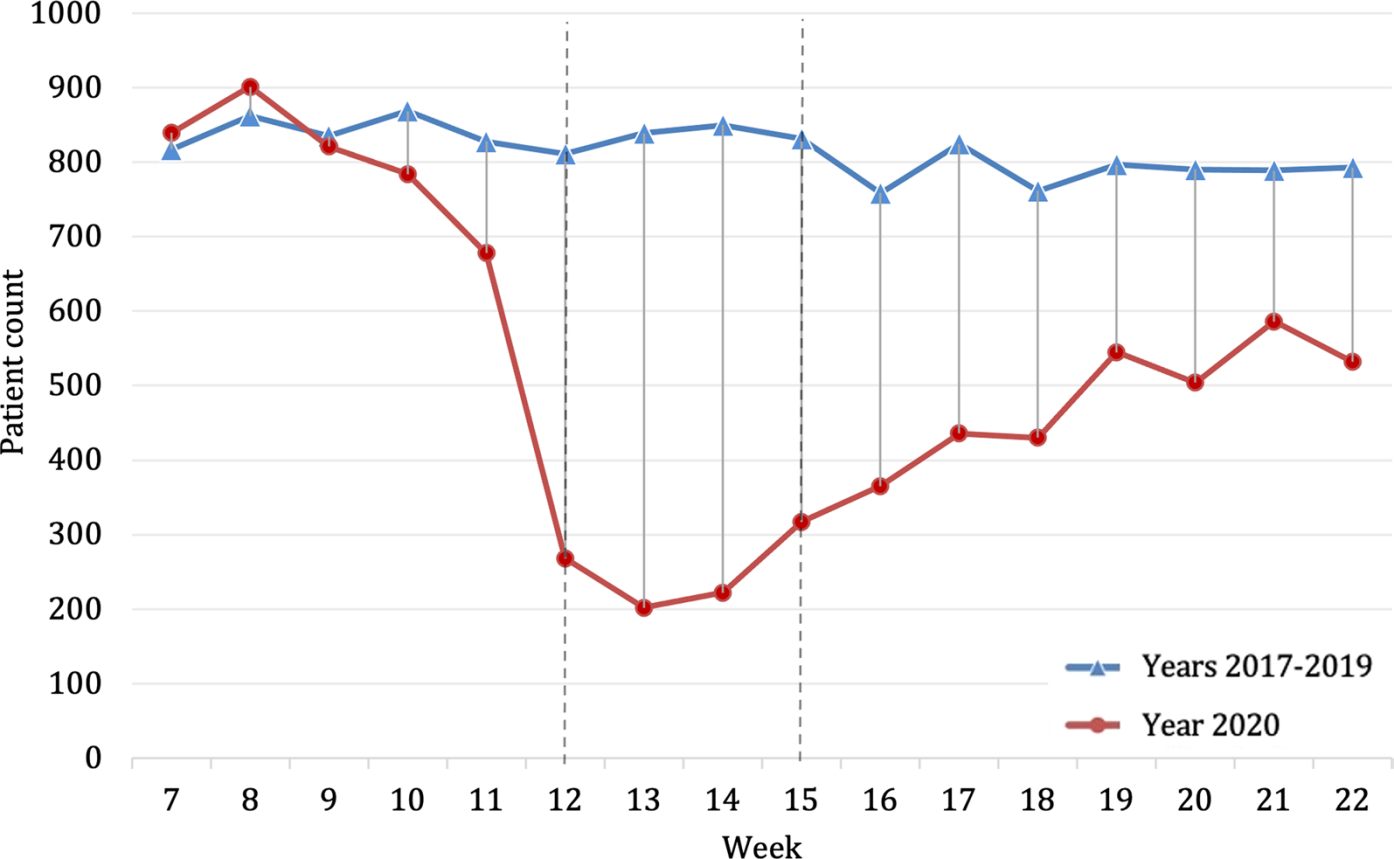
Number of patients admitted to the trauma center per week within the study period weeks 7–22; mean presentations per week in 2017–2019 (blue, triangle) compared to the presentations per week in 2020 (red, circle). Beginning and end of lockdown are marked with lines in weeks 12 and 15

Looking at the socio-demographic data, overall, no significant difference regarding age, gender distribution and country of residence could be observed when comparing the year of 2020 against the previous years (Table 1). However, during the lockdown in weeks 12–15 of 2020, a significant decrease in patients residing outside of Austria (e.g. Germany or other countries) (2020, *n* = 6; 2017–2019, *n* = 715; p < 0.001) being treated was noticeable. During the lockdown period, the number of patients decreased significantly, reaching the lowest point in week 13. Comparing average admissions—per week, a decrease of 69.7% (Fig. 1) in the lockdown period of 2020 (*n* = 252.3) compared to the previous years (*n* = 832.8) could be shown.

**Table 1:** Sociodemographic characteristics of study participants in 2020, broken down by period. Sociodemographic characteristics of study participants in 2017–2019, broken down by year

	2020 (*n* = 8430)				2017–2019 (*n* = 39,160)				Comparison of total (p-value)
	Total	Pre-lockdown (week 7–11)	Lockdown (week 12–15)	Post-lockdown (week 16–22)	Total	2019 (*n* = 12,772)	2018 (*n* = 13,318)	2017 (n = 13,070)	
Age in years ^a,^	40.9 ± 24.8	39.9** ± **23.9	42.0 ± 26.8	41.7 ± 25.3	40.6 ± 24.0	40.4 ± 24.2	40.6 ± 24.0	40.8 ± 23.7	0.331
Male gender ^b^	4770 (56.6)	2329 (57.9)	587 (58.2)	1854 (54.6)	22,111 (56.5)	7181 (56.2)	7445 (55.9)	7485 (57.3)	0.840
Country of residence ^b^	**–**				**–**				
Austria	7817 (92.7)	3,449 (85.7)	1003 (99.4)	3365 (99.0)	36,090 (92.2)	11,766 (92.1)	12,274 (92.2)	12,050 (92.2)	0.077
Germany	280 (3.3)	265 (6.6)	3 (0.3)	12 (0.4)	1448 (3.7)	480 (3.8)	495 (3.7)	473 (3.6)	0.094
Others	333 (4.0)	309 (7.7)	3 (0.3)	21 (0.6)	1622 (4.1)	526 (4.1)	549 (4.1)	547 (4.2)	0.421

Bold was to demark “total” from the subcategories following

Comparison of the 2020 total and the 2017–2019 total during the study period

^a^mean ± standard deviation

^b^absolute number (percent)

**Fig. 2:**
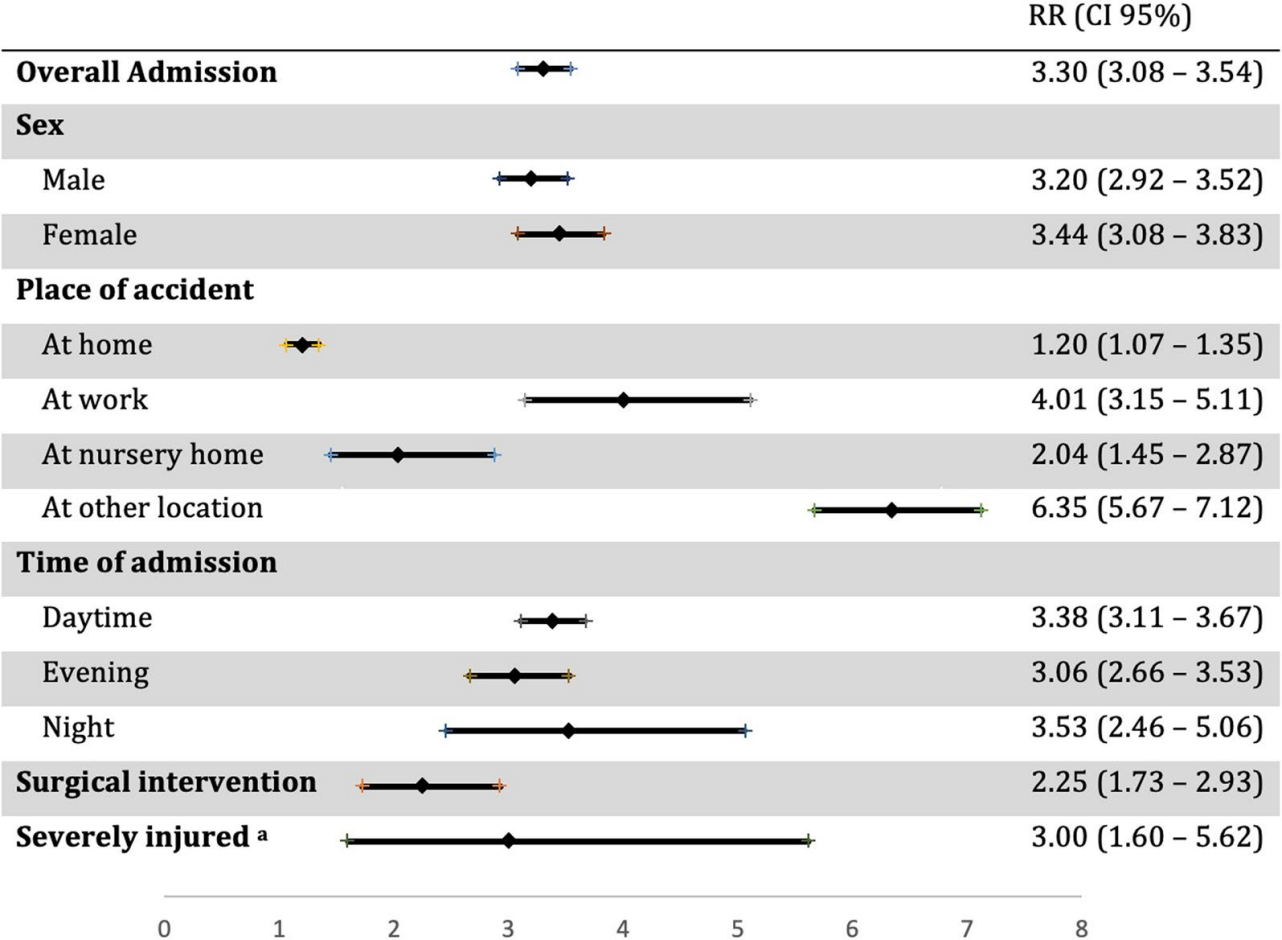
Incidence of trauma admissions within the period of the strict lockdown, comparing previous years with 2020 in specific subgroups. RR = relative risk, CI = confidence interval. ^a^Severely injured patients were assessed with New Injury Severity Scale > 12

During the corresponding weeks of lockdown, the relative risk of admission was 3.3 times higher in previous years than in 2020. A trend could be observed for women to be more likely admitted compared to males in previous years (Fig. 2).

### Place of accident

**Fig. 3:**
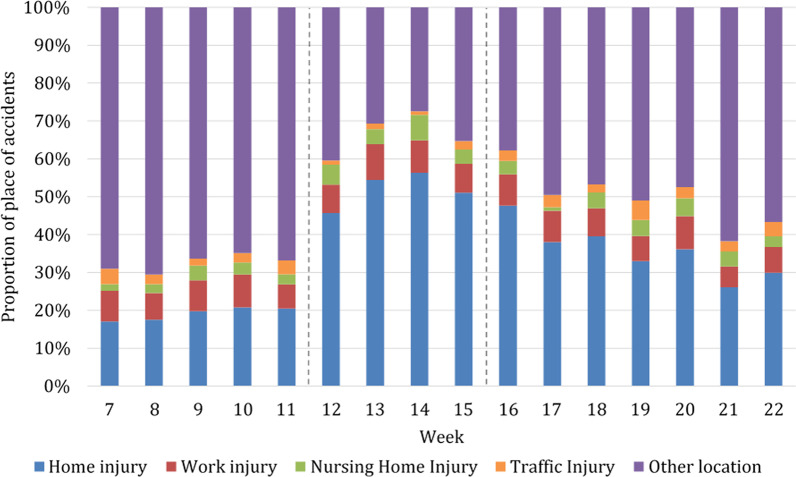
Proportion of all patients admitted to the trauma center per week within the study period in the year 2020 depending on the place of accident. Beginning and end of lockdown are marked with lines in weeks 12 and 15

While in earlier years the relative amount of place of accidents has remained constant, we observed a noticeable shift over the study period in 2020. In 2020, we observed an increase in the percentage of accidents at home during the lockdown, while injuries at other locations (e.g., injuries in the mountains, injuries caused by animals, etc.) decreased (Fig. 3).

### Accidents at home

**Fig. 4:**
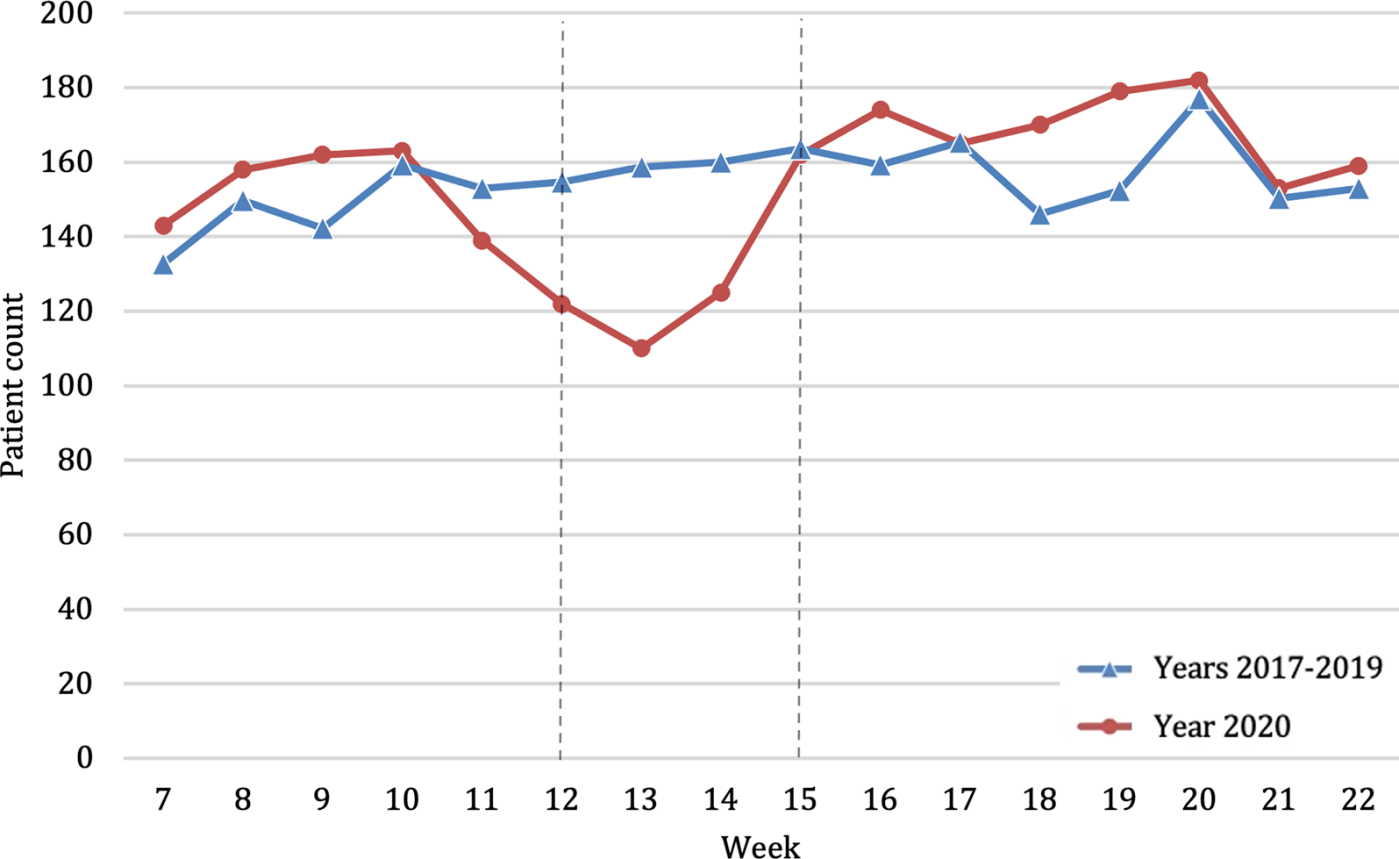
Absolute number of patients admitted because of place of accident at home throughout the study period comparing years 2017–2019 (blue, triangle) with the year 2020 (red, circle). Beginning and end of lockdown are marked with lines in weeks 12 and 15

Accidents at home decreased by 18.5% during the lockdown in 2020 compared to previous years (2020, n average per week = 129.8; 2017–2019, n average per week = 159.6; *p* < 0.001) (Fig. 4). However, these accidents at home represented 52.1% of all  places of accidents during the lockdown (Fig. 3), compared to only 18.9% during the same weeks in the earlier years (Table 2). The relative risk of admission was 1.2 times higher in previous years compared to 2020 during lockdown period (Fig. 2). Home injuries increased after the lockdown to such an extent that they exceeded the absolute numbers of the average of the previous years (Fig. 4).

**Table 2:** Absolute and relative count of patients according to the place of accident, severely injured patients, number of surgical interventions, in 2020 and average of the years 2017–2019 per week [absolute number (percent)]

	2020 (*n* = 8430)			2017–2019 (*n* = 39,160)		
	Pre-lockdown (week 7–11)	Lockdown (week 12–15)	Post-lockdown (week 16–22)	Week 7–11	Week 12–15	Week 16–22
*Place of accident*						
At home	786 (19.5)	536 (52.1)	1196 (35.2)	2162 (17.1)	1893 (18.9)	3250 (19.7)
At work	309 (7.7)	82 (8.1)	246 (7.2)	1194 (9.5)	988 (9.9)	1696 (10.3)
At a nursing home	111 (2.8)	49 (4.9)	120 (3.5)	362 (2.9)	301 (3.0)	514 (3.1)
Traffic accidents	116 (2.9)	15 (1.5)	112 (3.3)	314 (2.5)	347 (3.5)	719 (4.3)
Severely injured	59 (1.6)	13 (1.4)	47 (1.5)	184 (1.6)	118 (1.3)	175 (1.2)
Number of surgical interventions	256 (6.4)	80 (7.9)	223 (6.6)	748 (5.9)	539 (5.4)	724 (4.4)

### Accidents at work

**Fig. 5:**
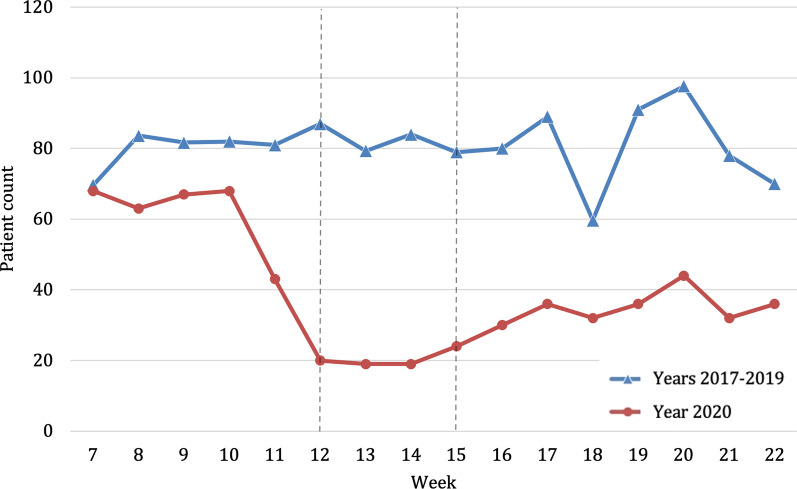
Number of patients admitted because of an accident at work throughout the study period comparing years 2017–2019 (blue, triangle) with year 2020 (red, circle). Beginning and end of lockdown are marked with lines in weeks 12 and 15

Accidents at work decreased by 75.1% during the lockdown in 2020 compared to previous years (2020, *n* average per week = 20.5; 2017–2019, n average per week = 82.3, *p* = 0.072) (Fig. 5). However, accidents at work represented 8.1% of all locations in the weeks of the hard lockdown in 2020 (Fig. 3) compared to 9.9% in previous years (Table 2). In this regard, the relative risk of admission with a work injury was four times higher in previous years (Fig. 2). After the end of the hard lockdown these injuries remained at a lower percentage level (7.2%) compared to the corresponding weeks in previous years (10.3%).

### Accidents at nursing homes

**Fig. 6:**
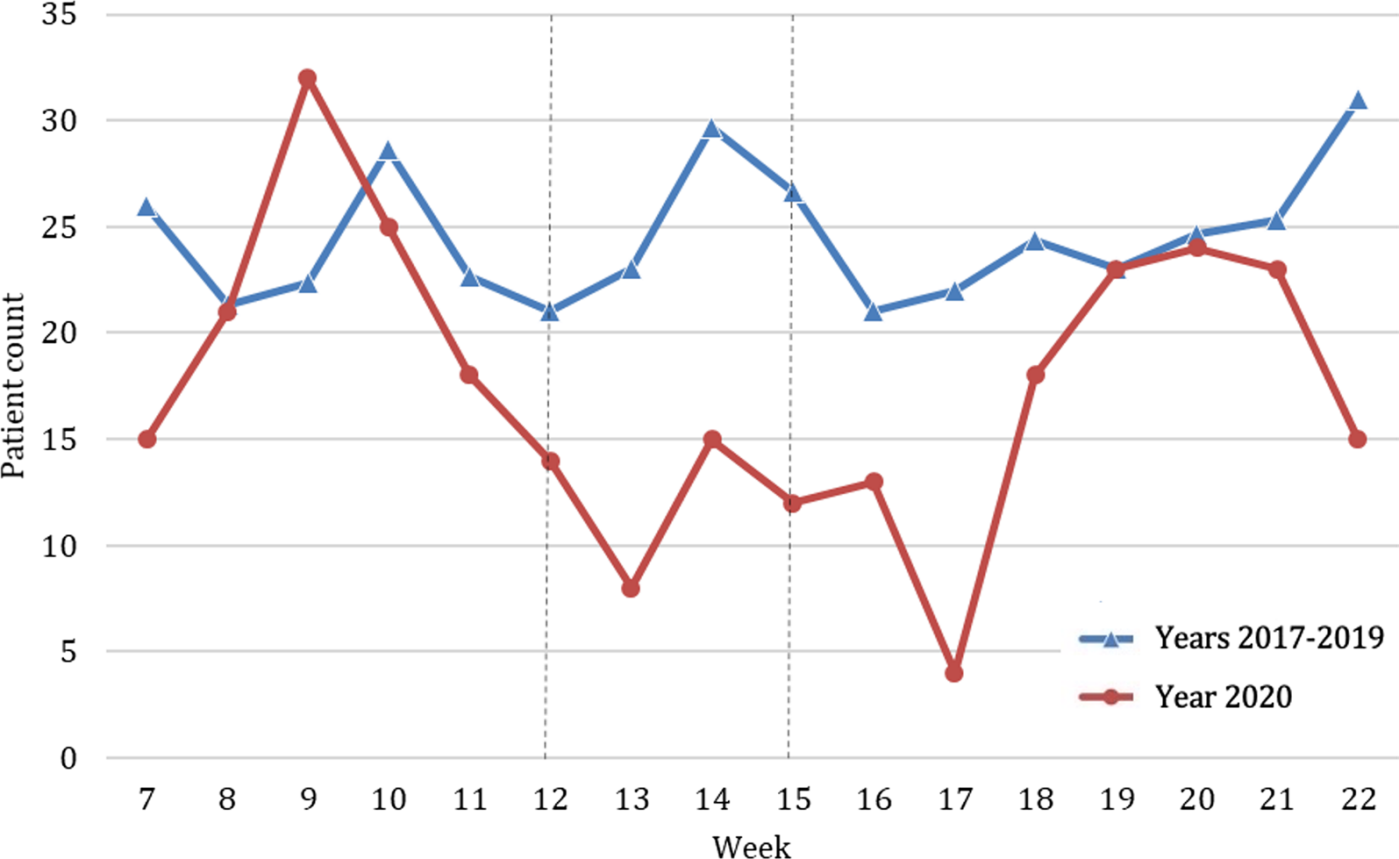
Number of patients admitted because of an accident at a nursing home throughout the study period comparing years 2017–2019 (blue, triangle) with year 2020 (red, circle). Beginning and end of lockdown are marked with lines in weeks 12 and 15

During the weeks of the strict lockdown in 2020 there was a reduction by 51.2% in numbers of patients who injured themselves in their nursing home (2020, *n* average per week = 12.3; 2017–2020, n average per week = 25.1, *p* = 0.001) (Fig. 6). However, the proportion of these injuries remained similar in 2020 during the lockdown (4.9%) to previous years (3.0%) (Table 2). In this period, the relative risk of admission was two times higher in previous years (Fig. 2).

### Traffic accidents

**Fig. 7:**
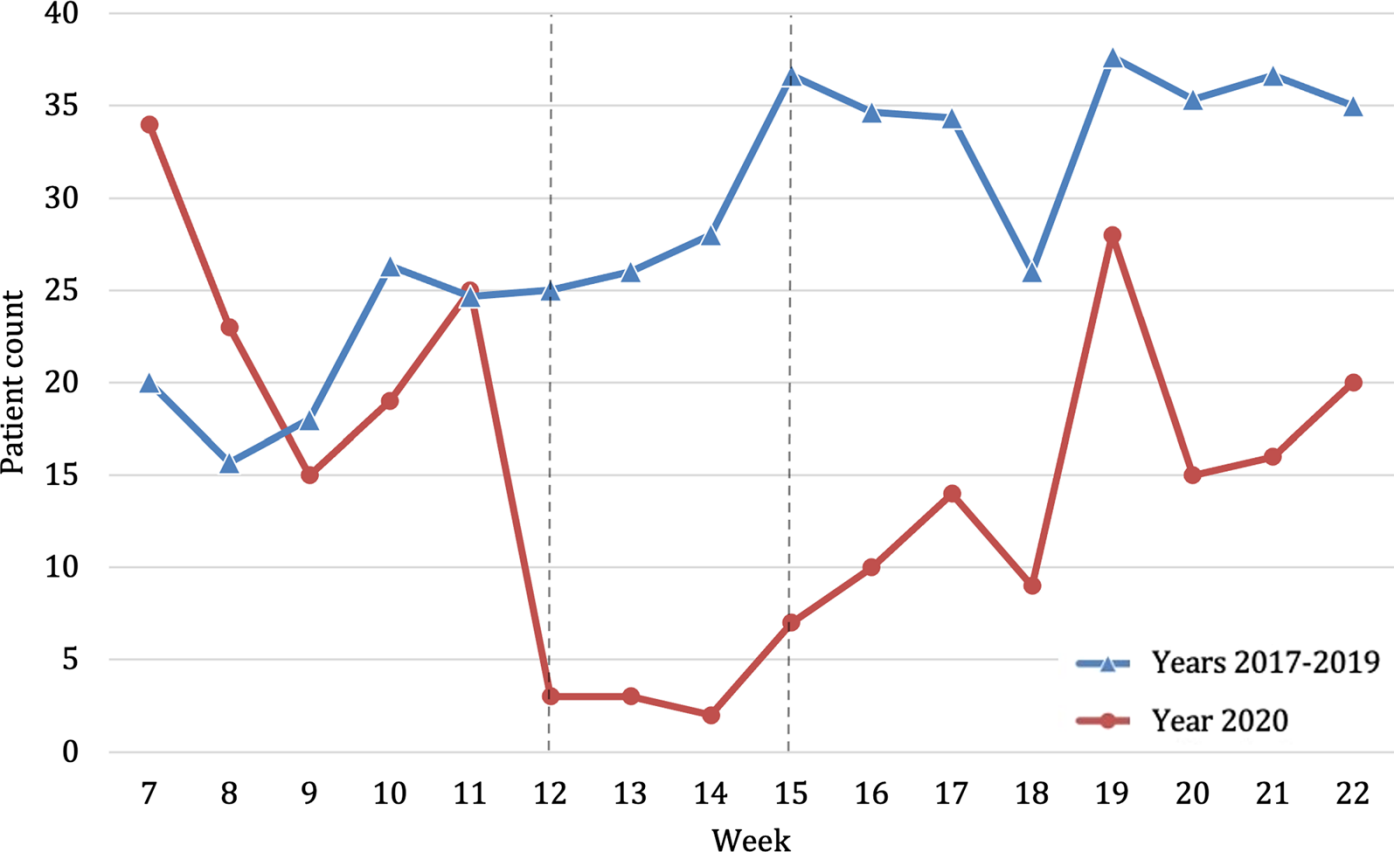
Number of patients admitted because of a traffic accident throughout the study period comparing years 2017–2019 (blue, triangle) with year 2020 (red, circle). Beginning and end of lockdown are marked with lines in weeks 12 and 15

During the lockdown in 2020 the number of patients admitted because of a traffic accident was reduced by 87.0% (2020, *n* average per week = 3.8; 2017–2019, *n* average per week = 28.9, *p* = 0.001) (Fig. 7). Traffic accidents represented 1.5% of all locations in the weeks of the hard lockdown in 2020 compared to 3.5% in previous years (Fig. 3, Table 2). The relative risk of admission was 7.7 times higher in previous years, which was the highest relative risk of admission of all evaluated variables (RR 7.73; 95% CI 4.52–13.24).

### Severity of injury

**Fig. 8:**
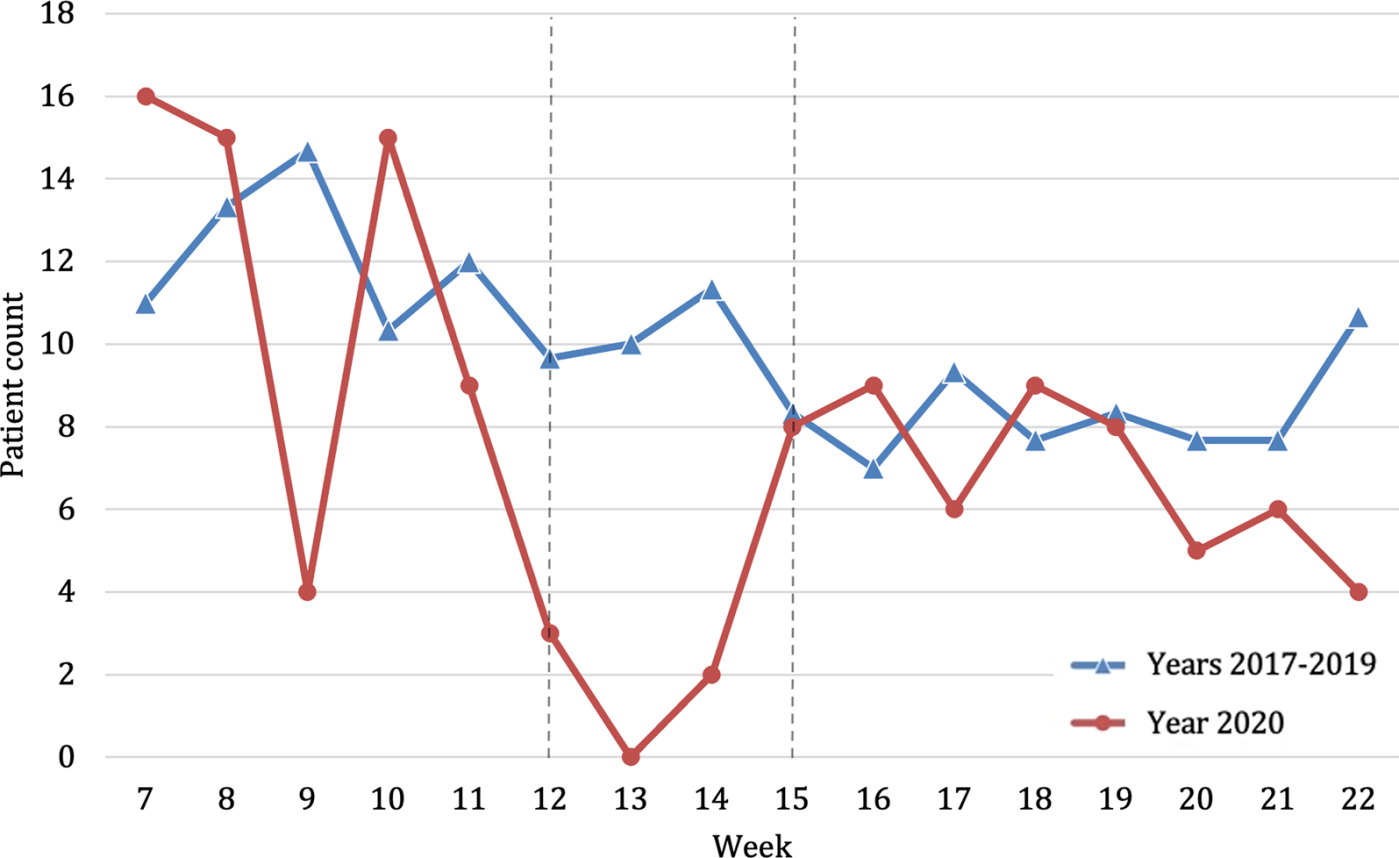
Number of severely injured patients admitted to the trauma center according to the New Injury Severity Scale in 2020. Beginning and end of lockdown are marked with lines in weeks 12 and 15

Rating all injured patients with the NISS, a decrease of 66.9% in patients coded as severely injured (NISS > 12) in the weeks of the lockdown in 2020 compared to previous years was noticeable (2020, n average per week = 3.3; 2017–2019, n average per week = 9.8, *p* = 0.760) (Fig. 8). However, the proportion of severely injured patients remained similar with 1.4% of all injuries in the weeks of strict lockdown in 2020 compared to 1.3% in previous years (Table 2). The relative risk of severe injury was three times higher in previous years than in 2020 during lockdown period.

### Surgical intervention

**Fig. 9:**
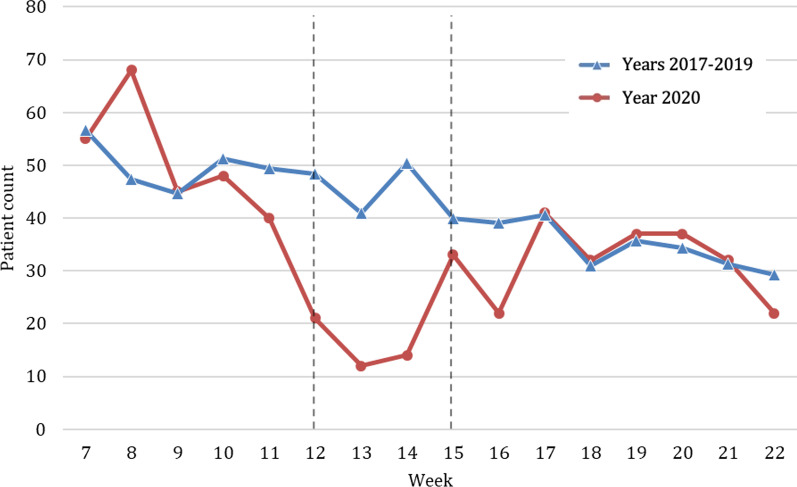
Number of patients admitted to the trauma center undergoing surgery comparing years 2017–2019 (blue, triangle) with year 2020 (red, circle). Beginning and end of lockdown are marked with lines in weeks 12 and 15

During the weeks of lockdown, we observed a reduction of 55.5% of patients undergoing surgery (2020, *n* average per week = 20; 2017–2019 = 44.9, *p* = 0.001) (Fig. 9). Of those admitted to trauma center the relative amount being treated by surgical intervention was 7.9% in 2020 compared to 5.4% in previous years 2017–2019 (Table 2). The relative risk was 2.3 times higher in previous years during the weeks of the lockdown (Fig. 2). After the lockdown ended, there was an increase in these patients reaching in absolute numbers almost the average of 2017–2019 (2020, *n* average per week = 31.9; 2017–2019 = 34.5). In this context, the percentage of patients admitted to the trauma center undergoing surgery after lockdown in 2020 was considerably higher (6.6%) than in previous years (4.4%) (Table 2).

### Time of treatment

**Fig. 10:**
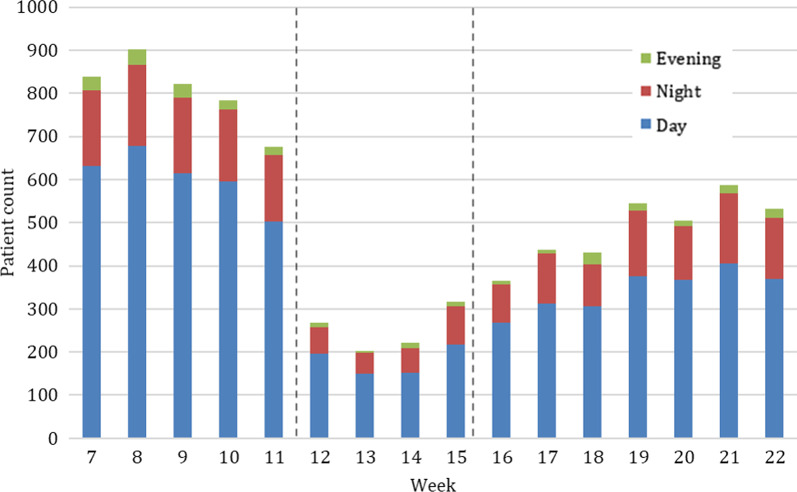
Number of patients admitted to the trauma center in 2020 throughout the study period grouped into admissions at day, evening and night. Beginning and end of lockdown are marked with lines in weeks 12 and 15

During the weeks of strict lockdown in 2020 the number of patients admitted during the night decreased by 71.6% (2020, *n* per average = 9.5; 2017–2019, *n* per average = 33.5, *p* = 0.692) (Fig. 10). However, the proportion of patients treated at night remained similar with 3.8% of all injuries in the weeks of strict lockdown in 2020 compared to 4.0% in previous years. The relative risk of admission at night was 3.5 times higher in previous years (Fig. 2).

### Body region of injury

**Fig. 11:**
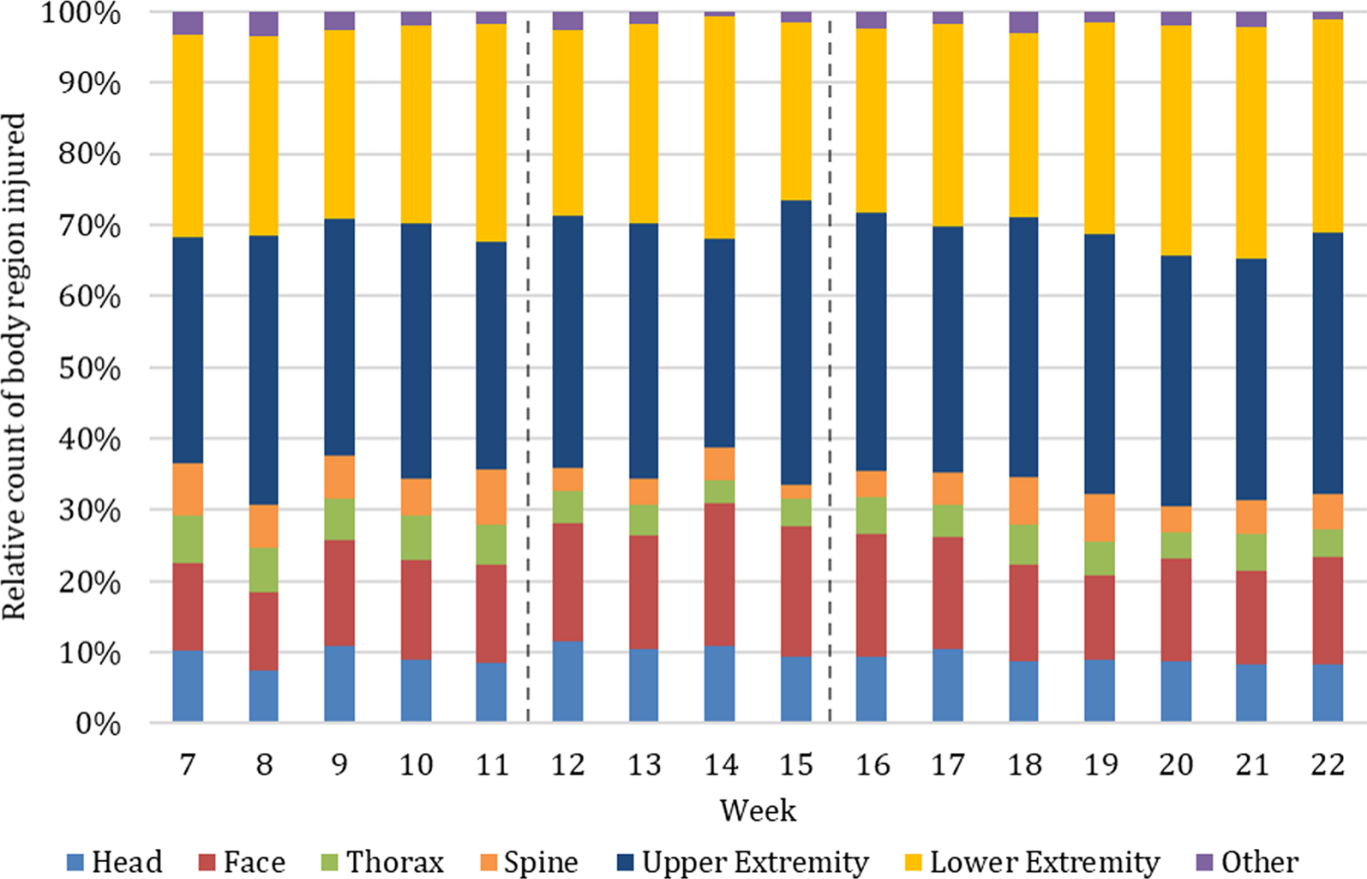
Relative count of body region injured in 2020. Beginning and end of lockdown are marked with lines in weeks 12 and 15

Concerning the body region of injury, 10,432 injuries were evaluated in 2020 and 44,977 in 2017–2019. The majority of these injuries, across all injuries both in 2020 and in previous years, involved the upper (> 29%) and lower extremities (> 25%) (Table 3). The proportion of head and face injuries increased the most during the lockdown period in 2020 (28.3%) (Fig. 11) in comparison with previous years (21.0%). The proportion of injuries to the thorax and spine decreased the most during lockdown in 2020 (6.4%) compared to previous years (10.5%). Other injuries including injuries to the neck, abdomen and external (e.g., hypothermia) remained similar.

**Table 3:** Absolute and relative count of injuries according to the body region and anatomical structure injured in 2020 and average of the years 2017–2019 per week [absolute number (percent)]

	2020 (*n* = 10,432)			2017–2019 (*n* = 44,977)		
	Pre-lockdown (week 7–11)	Lockdown (week 12–15)	Post-lockdown (week 16–22)	Week 7–11	Week 12–15	Week 16–22
*Body region*						
Head and Face	216 (22.4)	89.5 (28.3)	142.6 (23.1)	204.7 (21.4)	199.9 (21.0)	182.2 (20.0)
Thorax and Spine	121.2 (12.5)	20.3 (6.4)	59.7 (9.7)	112.9 (11.8)	100.5 (10.5)	89.2 (9.8)
Upper and Lower Extremity	604.2 (62.4)	199.0 (62.8)	401.1 (65.0)	612.0 (63.9)	626.1 (65.7)	613.0 (67.2)
Other	26.8 (2.8)	5.5 (1.7)	14.1 (2.3)	37.8 (2.9)	27.0 (2.8)	28.2 (3.1)
*Anatomical structure*						
Superficial, Penetrating	476.4 (49.2)	192.3 (60.7)	348.1 (56.4)	468.4 (48.9)	497.1 (52.1)	502.6 (55.0)
Organs, Muscle, Tendon, Ligament	155.0 (16.0)	27.8 (8.8)	67.4 (10.9)	151.1 (15.8)	137.7 (14.4)	118.3 (13.0)
Skeletal and Joint	312.2 (32.2)	93 (29.4)	192.0 (31.1)	314.4 (32.8)	299.5 (31.4)	277.2 (30.4)
Other	25.2 (2.6)	3.8 (1.2)	9.7 (1.6)	23.6 (2.5)	15.5 (1.6)	15.0 (1.6)

### Injured anatomical structures

**Fig. 12:**
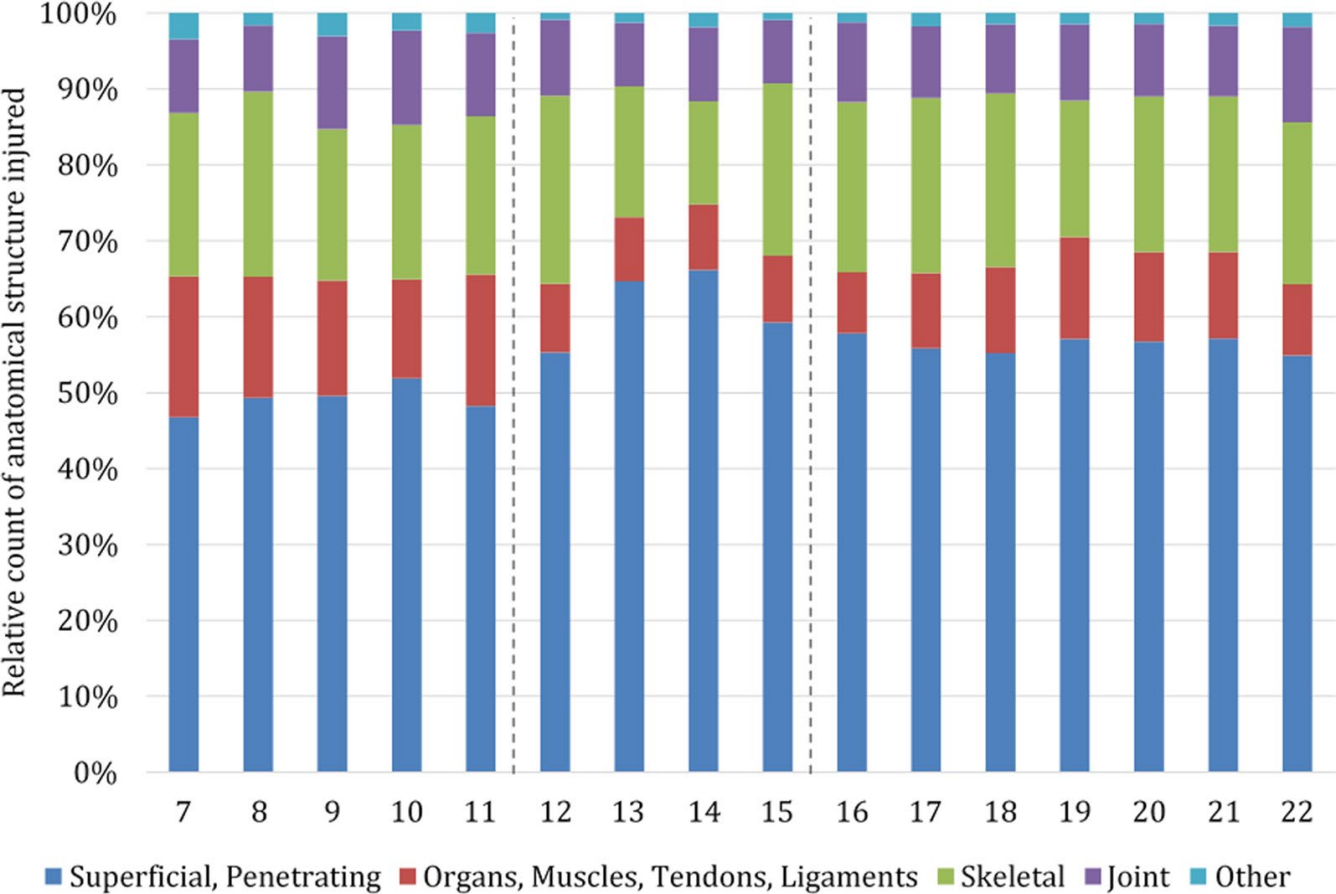
Relative count of anatomical structure injured in 2020. Beginning and end of lockdown are marked with lines in weeks 12 and 15

Concerning the injured anatomical structures, 10,432 injuries were evaluated in 2020 and 44,977 in 2017–2019. The relative count of all injured anatomical structures admitted in the year 2020 is shown in Fig. 12.

The majority of injuries, both in 2020 and in previous years, involved superficial and penetrating injuries (> 48%) (Table 3). In the weeks of lockdown, the proportion of superficial and penetrating injuries increased most with 60.7% in 2020 compared to 52.1% in previous years (2020, *n* average per week = 192.3; 2017–2019, *n* average per week = 497.1).

The proportion of injuries concerning “organs, muscles, tendons and ligaments” decreased most during the lockdown with 8.8% in 2020 compared to 14.4% in previous years. The proportion of skeletal and joint injuries remained similar with 29.4% during the lockdown in 2020 compared to 31.4% in previous years. Injuries to “other” anatomical structures (nerves, vessels, head/ loss of consciousness) remained similar with 1.2% in 2020 during lockdown compared to 1.6% in previous years.

## Discussion

The most important finding in this study was an overall reduction of 69.7% of patients admitted to the Trauma Emergency Ward in Innsbruck during the lockdown 2020. The greatest reduction of patients was seen in patients residing outside of Austria, patients treated at night, patients injured to the thorax or spine as well as severely injured patients. The greatest increase of patients was seen in patients injured at home, with injuries to the head or face as well as superficial and penetrating injuries.

As it was not allowed to leave the municipality of the registered place of residence during the aforementioned time, it seems natural that fewer non-resident patients had to be treated. Additionally, the country borders and hotels were closed, so tourism had to close down. This also might explain the decrease of these numbers. The dramatic decrease in number of patients who had to be treated at night can be attributed, amongst other factors, to the restrictions on movement and the closure of bars and restaurants. The drastic reduction in traffic accidents is in line with current data [5] and explained by the restrictions of mobility during the lockdown period. This could be a reason for the reduced injuries to the thorax and spine [6–9]. However, the ban on sports, in particular the ban on mountain sports, may also account for a significant decline in these injuries. Restricting traffic and sports is also likely to play a major role in the dramatic decrease in seriously injured patients.

Conversely, the proportion of home injuries increased significantly during lockdown, which is plausible, as people had to stay and work at home. Home injuries are often caused by falling or being cut or pierced by some objects [10]. This may explain the increase in head or face injuries as well as superficial or penetrating injuries during lockdown. Several studies in various countries have shown that home accidents have increased dramatically during periods of lockdown [11–13]. Accidents at home are therefore a definitive danger in times of a lockdown and should therefore be addressed in prevention campaigns.

Interestingly the absolute number of nursery home injuries decreased during lockdown compared to previous years. In this regard, comparative evidence in the recent literature is inconclusive. A study in Scotland showed no significant decrease in the number of treated fragility fractures, during the pandemic [14]. In contrast, two Italian and one Spanish study demonstrated a reduced volume of hip fractures during periods of lockdown [15–17]. It remains unclear as to why we observed a decrease in the absolute number of admissions from nursery homes in our study. In less severe injuries the fear of contracting COVID-19 may have prevented presentation to the trauma center [15]. An additional explanation for the decrease might be that minor injuries, such as small wounds, may have been treated by the nursing staff in the nursing home itself. The temporary restriction of visits to nursing homes, the limitation of the mobility of nursing home residents and the reduction of group activities during lockdown could, however, worsen the fragility of these patients in the long term [15]. A possible delaying long-term effect of fragility fractures should be evaluated in future studies.

It would also be worthwhile to analyze how patterns and volumes of injuries change depending on lockdown restrictions especially the ban on sports during only the lockdown evaluated in this study is likely to have had a strong impact on the above-mentioned results. But also, the willingness of the population to comply with restrictive rules during the first lockdown is likely to have been significantly higher than in the subsequent lockdowns. It would therefore be interesting to compare lockdown periods with the respective regulations.

During the hard lockdown in this study, the relative count of patients admitted to our trauma center undergoing surgery increased. This increase occurred even though elective surgeries were postponed in Austria during the lockdown, so only emergency surgeries were performed. Furthermore, many patients with injuries that, under normal circumstances, would have been treated surgically were treated conservatively in times of lockdown [18]. Guidelines recommend the use of removable splints and bracing whenever possible during the hard lockdown with limited surgical capacities [19]. In the future, there will be an immediate necessity to evaluate the impact on patient outcomes when surgical therapy is postponed.

One limitation of this study was its retrospective and single-center design. We can only report on the catchment area of the trauma center evaluated. A comparison of our data with peripheral hospitals would therefore be an important addition in future studies. Another weakness is the seasonal bias due to the time of year in the study period. In the strong tourism periods of the winter season, the impact of a lockdown is certainly greater than in the off-season of ski tourism. One strength of this study is the large number of patients and injuries evaluated and the comparison with the three previous years. This means that the number of outliers per year could be minimized.

## Conclusion

This study shows a significant change in patient count and injury patterns and number of patients admitted to a level one trauma center during and after a hard lockdown. In terms of awareness and injury prevention, interventions to reduce the risk of home injuries need to be focused on. As resources and capacities are limited during a hard lockdown, specific guidelines for conservative and operative management should be developed for each medical specialty, especially for trauma surgery.

## Data Availability

Data available on request from the authors.
